# Effectiveness of oral cephalexin in antibiotic-course completion for methicillin-susceptible *Staphylococcus aureus-*induced bacteremic vertebral osteomyelitis

**DOI:** 10.1186/s12879-023-08266-0

**Published:** 2023-05-08

**Authors:** Nobumasa Okumura, Kayoko Hayakawa, Kei Yamamoto, Gen Yamada, Kazuhisa Mezaki, Norio Ohmagari

**Affiliations:** 1 Disease Control and Prevention Center, National Center for Global Health and Medicine, 1-21-1 Toyama, Shinjuku-Ku, Tokyo, Japan; 2 Microbiology Laboratory, National Center for Global Health and Medicine, Tokyo, Japan

**Keywords:** Vertebral osteomyelitis, Oral therapy, Cephalexin

## Abstract

**Background:**

Methicillin-susceptible *Staphylococcus aureus* (MSSA) is the most common causative microorganism of pyogenic vertebral osteomyelitis (PVO). Although oral antimicrobial therapy with first-generation cephalosporins can treat MSSA infection, data on PVO are scarce. This study evaluated the treatment efficacy of cephalexin as oral antibiotic therapy for MSSA-induced PVO.

**Methods:**

This retrospective study included adult patients treated with oral cephalexin as the completing treatment for PVO with MSSA bacteremia from 2012 to 2020. Treatment effectiveness of cephalexin was evaluated by comparing improvement (5-point scale; score ≥ 4/5 indicates treatment success) in symptoms and laboratory and imaging results between intravenous antimicrobial and oral cephalexin treatment.

**Results:**

Among 15 participants (8 [53%] women; median [interquartile range, IQR], age 75 [67.5–80.5] years; Charlson Comorbidity Index 2 [0–4]), 10 (67%) had lumbar spine lesions, 12 (80%) had spinal abscesses, and 4 (27%) had remote abscesses; no patients had concomitant endocarditis. In 11 patients with normal renal function, cephalexin 1,500–2,000 mg/day was administered. Five patients (33%) underwent surgery. Median (IQR; range) duration (days) of intravenous antibiotics, cephalexin, and total treatment was 36 (32–61; 21–86), 29 (19–82; 8–251), and 86 (59–125; 37–337), respectively. Cephalexin had an 87% treatment success rate without recurrence during a median follow-up of 119 (IQR, 48.5–350) days.

**Conclusions:**

In patients with MSSA bacteremia and PVO, antibiotic treatment completion with cephalexin is a reasonable option, even in cases with spinal abscess, if at least 3 weeks of effective intravenous antimicrobial therapy is provided.

## Background

*Staphylococcus aureus* is the most common causative microorganism of pyogenic vertebral osteomyelitis (PVO) [1]. The standard treatment duration for PVO is 6 weeks [1], and intravenous antibiotics are not usually required for the entire treatment duration. As the early transition from intravenous to oral antimicrobial agents does not worsen the prognosis in patients with osteoarticular infections [2], this approach is being applied to the management of patients with PVO. Levofloxacin plus rifampicin or linezolid are recommended oral antimicrobial agents for methicillin-susceptible *S. aureus* (MSSA)-induced PVO [1]. However, oral first-generation cephalosporins, which are widely used as first-line agents for non-PVO MSSA infections, should also be considered in PVO to prevent drug resistance and minimize side effects. Cephalexin is the leading oral first-generation cephalosporin; however, there is limited data on oral cephalexin treatment for MSSA-induced PVO. This study was conducted to determine the usefulness of switching to oral cephalexin as antibiotic therapy for MSSA-induced PVO.

## Methods

### Study design and participants

This single-center, retrospective, descriptive study in Japan enrolled patients aged ≥ 18 years diagnosed with PVO and MSSA-induced bacteremia and used cephalexin for at least one week between April 2012 and March 2021 at the National Center for Global Health and Medicine Hospital. MSSA-induced bacteremia was defined based on the detection of *S. aureus* with a minimum inhibitory concentration of oxacillin ≤ 2 μg/mL in at least one set of blood cultures. PVO was diagnosed on fulfillment of all three of the following criteria: 1) clinical findings: back pain, neurologic symptoms, or fever; 2) laboratory findings: elevated C-reactive protein (CRP) or erythrocyte sedimentation rate (ESR); and 3) imaging findings: abnormal findings in the spine on computed tomography (CT) or magnetic resonance imaging (MRI). Cases with polymicrobial infections or insufficient medical record data were excluded from this study.

### Data collection

Data on demographics, comorbidities, and complications; clinical, laboratory, and radiographic findings; blood culture results, surgical treatment, and antimicrobial therapy; and clinical course and outcomes were collected by chart review of electronic medical records. The Charlson Comorbidity Index (CCI) [3] was calculated to determine the influence of comorbidities. The affected vertebrae were counted at the cervical, thoracic, lumbar, and sacral levels, and duplicated counts were allowed in patients with multiple affected sites. Complications were evaluated for 1) paravertebral, epidural, and iliopsoas abscesses, 2) abscesses in other organs, and 3) infective endocarditis. For antimicrobial therapy, we calculated the duration of total antimicrobial, intravenous antimicrobial, oral antimicrobial, and cephalexin therapies. Patients who required indefinite chronic suppressive therapy were excluded from the subgroup analyses of the duration of total antimicrobial, oral antimicrobial, and cephalexin therapies. Data were extracted for all types of antimicrobial agents used for at least 1 day.

### Efficacy evaluation

Treatment efficacy was determined at two time points: at the end of intravenous antimicrobial therapy (compared with that at treatment initiation) and at the end of cephalexin therapy (compared with that after intravenous antimicrobial therapy). In cases where chronic suppressive therapy was used, we used the median time point of the total treatment duration for all cases except those in which chronic suppressive therapy was used instead of the time point of cephalexin termination. Based on previous reports [1, 4, 5], a 5-point scale (5—definite success, 4—probable success, 3—inconclusive, 2—probable failure, and 1—definite failure) was developed based on clinical, laboratory, and radiographic findings to determine the treatment response (Table 1). Clinical findings were evaluated according to three levels of pain symptoms: improving, unchanged, and worsening. Laboratory findings were evaluated by three levels of CRP: improving (≥ 25% decrease), unchanged (changes not meeting either of the other two criteria), and worsening (increase above the upper limit of normal, 0.14 mg/dL and > 25% increase) [5]. Radiographic findings were evaluated based on the radiologist’s report on a four-point scale: improving, unchanged, worsening, and unknown. Score ≥ 4/5 was considered treatment success. Moreover, relapse, diagnosed by clinical judgment within 1 year after cephalexin termination, was evaluated.

**Table 1:** Evaluation of treatment efficacy for vertebral osteomyelitis using a 5-point scale

Pain	CRP	Comorbid conditions, besides vertebral osteomyelitis, that can increase CRP	Imaging	Judgment	Score
< At the end of IV antimicrobial therapy >					
Improving	Improving		Improving	Definite success	5
			Unknown	Probable success	4
			Unchanged	Probable success	4
			Worsening	Inconclusive	3
	Unchanged/worsening	Yes	Improving	Probable success	4
			Unknown	Inconclusive	3
			Unchanged	Inconclusive	3
			Worsening	Probable failure	2
		No	Improving	Probable success	4
			Unknown	Inconclusive	3
			Unchanged	Inconclusive	3
			Worsening	Probable failure	2
Unchanged/worsening	Improving		Improving	Probable success	4
			Unknown	Inconclusive	3
			Unchanged	Inconclusive	3
			Worsening	Probable failure	2
	Unchanged/worsening	Yes	Improving	Probable success	4
			Unknown	Inconclusive	3
			Unchanged	Inconclusive	3
			Worsening	Probable failure	2
		No	Improving	Inconclusive	3
			Unknown	Probable failure	2
			Unchanged	Probable failure	2
			Worsening	Definite failure	1
< At the end of cephalexin therapy >					
Improving/unchanged	Improving/unchanged		Improving	Definite success	5
			Unknown	Probable success	4
			Unchanged	Probable success	4
			Worsening	Inconclusive	3
	Worsening	Yes	Improving	Probable success	4
			Unknown	Inconclusive	3
			Unchanged	Inconclusive	3
			Worsening	Probable failure	2
		No	Improving	Probable success	4
			Unknown	Inconclusive	3
			Unchanged	Inconclusive	3
			Worsening	Probable failure	2
Worsening	Improving/unchanged		Improving	Probable success	4
			Unknown	Inconclusive	3
			Unchanged	Inconclusive	3
			Worsening	Probable failure	2
	Worsening	Yes	Improving	Probable success	4
			Unknown	Inconclusive	3
			Unchanged	Inconclusive	3
			Worsening	Probable failure	2
		No	Improving	Inconclusive	3
			Unknown	Probable failure	2
			Unchanged	Probable failure	2
			Worsening	Definite failure	1

### Statistical analysis

We described the patients’ characteristics using median and interquartile range (IQR) for continuous variables and number and percentage (%) for categorical variables. Efficacy scores were described in terms of the number and percentage of patients who received each score.

### Ethics statement

This study was approved by the ethics committee of the National Center for Global Health and Medicine prior to the study’s initiation (Approval number: NCGM-S-004301-00), and the requirement to obtain patient consent was waived.

## Results

### Study population

In the 9-year period from April 2012 to March 2021, there were 385 cases of MSSA bacteremia. Of these, 44 had coexisting PVO, and 16/44 cases received oral cephalexin treatment. In this study, 1 patient was excluded due to a polymicrobial infection (*Streptococcus agalactiae*), and 15 cases were included. Table 2 presents the participant characteristics of these 15 patients: 8 (53%) were female, the median age was 75 [IQR 67.5–80.5] years, the median weight was 57.0 [IQR 45.5–62.5] kg, the median BMI was 22.1 [IQR 19.8–24.0] kg/m^2^. Regarding comorbidities, 3 patients had a myocardial infarction, whereas ulcer disease, moderate or severe liver disease, diabetes with end-organ damage, congestive heart failure, and any tumor were seen in two patients each, and cerebrovascular disease, dementia, diabetes, moderate or severe renal disease, peripheral vascular disease, and metastatic solid tumor were seen in one patient each. The median CCI score was 2 [IQR 0–4]. None of the patients had a history of spinal surgery or spinal implants.

**Table 2:** Baseline characteristics of participants with bacteremic vertebral osteomyelitis due to methicillin-susceptible *Staphylococcus aureus* (*n* = 15)

Age, years	75 [67.5–80.5]
Female	8 (53%)
BMI^a^	22.1 [19.8–24.0]
Charlson Comorbidity Index	2 [0–4]
Site of vertebral osteomyelitis^b^	
Cervical level	3 (20%)
Thoracic level	5 (33%)
Lumbar level	10 (67%)
Sacral level	4 (27%)
Previous spinal implant placement	0 (0%)
Complications	
Endocarditis	0 (0%)
Spinal abscesses^c^	12 (80%)
Abscesses at remote sites^d^	4 (27%)

Data are median [IQR] or number (%) unless otherwise specified

^a^One case was excluded due to missing data

^b^Duplicate selections were allowed

^c^Includes paravertebral, epidural, and psoas abscesses

^d^Each case with a subcutaneous abscess of inguinal region (surgical site), piriformis abscess, posterior neck and erector spinae abscess, and anterior mediastinal abscess, respectively

The affected vertebrae were at the cervical, thoracic, lumbar, and sacral levels in 3 (20%), 5 (33%), 10 (67%), and 4 (27%) patients, respectively. Complications included paravertebral, epidural, or iliopsoas abscesses that occurred in 12 patients (80%) and abscesses of other organs in 4 patients (27%). Transthoracic echocardiography was performed in all 15 patients, 3 of whom underwent transesophageal echocardiography. The presence or absence of infective endocarditis was determined by an infectious disease physician based on physical examination and echocardiographic findings, and none of the patients had coexisting endocarditis. Follow-up blood cultures were obtained in all patients, and the median time to negative blood cultures was 6 [IQR 4–7] days.

### Antibiotic and surgical treatment

**Fig. 1:**
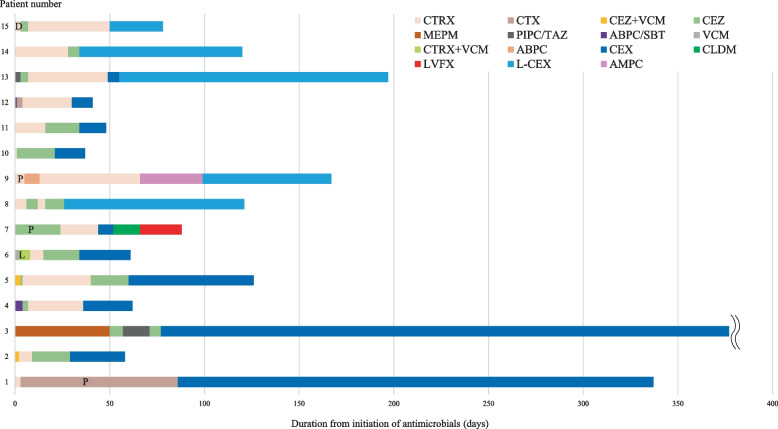
Type and duration of antimicrobials in all patients. #3 received chronic suppressive therapy. #4 died of aspiration pneumonia during treatment of PVO. #10 discontinued cephalexin due to a urinary tract infection. CTRX: ceftriaxone, CTX: cefotaxime, CEZ: cefazolin, VCM: vancomycin, MEPM: meropenem, PIPC/TAZ: piperacillin-tazobactam, ABPC/SBT: ampicillin-sulbactam, ABPC: ampicillin, CEX: cephalexin, CLDM: clindamycin, LVFX: levofloxacin, L-CEX: cephalexin complex granules, AMPC: amoxicillin, P: percutaneous abscess drainage, L: laminectomy, D: posterior decompression with fusion

The types of antimicrobial agents and their administration durations are shown in Fig. 1. The median [IQR and range] duration (days) of intravenous antimicrobial therapy, oral antimicrobial therapy, cephalexin administration, and total treatment was 36 [32–61 and 21–86], 40 [26–93 and 11–251], 29 [19–82 and 8–251], and 86 [59–125 and 37–337], respectively (Table 3). The most frequently used intravenous antibacterial agent was ceftriaxone (14 cases, 93%), followed by cefazolin (12 cases, 80%), ampicillin-sulbactam (3 cases, 20%), vancomycin (3 cases, 20%), piperacillin-tazobactam (2 cases, 13%), cefotaxime (2 cases, 13%), meropenem (1 case, 6.7%), and ampicillin (1 case, 6.7%). The intravenous antibacterial agent used for the longest duration for each patient was ceftriaxone (7 cases, 47%), cefazolin (6 cases, 40%), cefotaxime (1 case, 6.7%), meropenem (1 case, 6.7%). Cephalexin was used for the oral transition from intravenous antibacterial agents in 14 patients (93%), whereas amoxicillin was used in the remaining patient. A patient with a taste disorder, after 1-month of amoxicillin treatment, was switched to cephalexin. In 11 patients with normal renal function (Cockcroft–Gault creatinine clearance ≥ 50 mL/min) at the time of transition to oral antimicrobial therapy, the daily cephalexin dose was 2,000 (*n* = 9) and 1,500 mg (*n* = 2). The formulation used was cephalexin capsule in 10 patients and cephalexin complex granules (i.e., a mixture of gastro-soluble and enteric-coated preparations) in 5 patients. Cephalexin was not used in combination with other antimicrobial agents in any of the cases. Cephalexin was discontinued before completion of PVO treatment in 3 patients (20%): one patient was switched to clindamycin due to mild elevation of liver enzyme and eosinophilia; one was switched to intravenous cefepime due to a urinary tract infection, and one patient was on cephalexin treatment and died of aspiration pneumonia. The median length of hospital stay was 64 [IQR 45–81, range 27–101] days. The need for surgical treatment was ultimately determined by the orthopedic surgeon based on imaging findings. Five patients (33%) underwent surgery: percutaneous abscess drainage was done in 3 patients, laminectomy in 1 patient, and posterior decompression with fusion in 1 patient. Surgical treatment was required to reduce the volume of the iliopsoas abscess in three patients who underwent percutaneous abscess drainage and because of neurological symptoms in the lower extremities due to epidural abscess in two patients who underwent laminectomy or posterior decompression with fusion.

**Table 3:** Duration of antibiotic therapy and surgical treatment in bacteremic vertebral osteomyelitis due to methicillin-susceptible *Staphylococcus aureus* (*n* = 15)

Intravenous treatment duration, days	36 [32–61]
Oral treatment duration^a^, days	40 [26–93]
Cephalexin administration duration^a^, days	29 [19–82]
Discontinuation of cephalexin before treatment completion	3 (20%)^b^
Total treatment duration^a^, days	86 [59–125]
Surgical treatment	5 (33%)

Data are median [IQR] or number (%) unless otherwise specified

^a^One case was excluded due to chronic suppression

^b^One discontinuation due to hepatotoxicity and eosinophilia, one due to concomitant urinary tract infection, and one death due to aspiration pneumonia

### Treatment outcomes

The imaging modalities used to determine efficacy were CT only in 2 cases, MRI only in 4 cases, and CT and MRI in 9 cases. At the end of intravenous antimicrobial therapy, the treatment response score was 5 in 9 patients (60%) and 4 in 6 patients (40%), with a treatment success rate of 100% (Table 4). At the end of cephalexin treatment, the treatment response score was 5 in 1 patient (7%), 4 in 12 patients (80%), and 3 in 2 patients (13%), with a treatment success rate of 87%. One of the two patients with a score of 3 for the cephalexin course had cephalexin therapy terminated due to urinary tract infection. The other patient had a CRP level of 0.06 and 0.2 mg/dL at the end of intravenous antimicrobial therapy and cephalexin treatment, respectively, which was considered as worsening. During the treatment period of PVO, one death occurred, and this was in a patient who had been successfully treated with cephalexin but died of aspiration pneumonia. Four patients were followed up for 1 year after completion of the cephalexin course, and none of the patients relapsed within 1 year (Table 4). The median follow-up period after completion of the cephalexin course for all patients was 119 [IQR 48.5–350] days and none of the patients relapsed during the follow-up period. A death that occurred during the follow-up period was attributed to liver cirrhosis.

**Table 4:** Efficacy of cephalexin therapy in bacteremic vertebral osteomyelitis due to methicillin-susceptible *Staphylococcus aureus*

Efficacy score at the end of intravenous antibiotic therapy (*N* = 15)	
5	9 (60%)
4	6 (40%)
3	0 (0%)
2	0 (0%)
1	0 (0%)
Efficacy score at the end of cephalexin therapy^a^ (*N* = 15)	
5	1 (7%)
4	12 (80%)
3	2 (13%)
2	0 (0%)
1	0 (0%)
Relapse within 1 year (*N* = 4)	
	0 (0%)

Data are number (%) unless otherwise specified

^a^In one case with chronic suppression, efficacy was determined at the median timepoint of the total duration of treatment in the cases after excluding the one with chronic suppression

## Discussion

PVO is an osteoarticular infection that requires long-term antimicrobial therapy. In recent years, the early transition from intravenous to oral antimicrobial therapy has become a trend in osteoarticular infections. In a randomized controlled trial, Bernard et al. showed that 6-week antimicrobial therapy for PVO was non-inferior to 12-week antimicrobial therapy in terms of a confirmed cure rate at 1 year after therapy completion; both groups were switched to oral antimicrobials after a median duration of approximately 2 weeks of intravenous antimicrobial therapy [4]. The OVIVA trial showed that starting oral antimicrobials within 7 days of treatment initiation in various osteoarticular infections did not increase the treatment failure rate within 1 year compared to that in patients who used intravenous antimicrobials [2]. A recent retrospective cohort study by Marconi et al. showed that, in patients with PVO, starting treatment with only oral antimicrobials does not increase the risk of treatment failure [6]. Infectious Diseases Society of America guidelines [1] recommends linezolid or levofloxacin plus rifampicin as oral antimicrobial agents for MSSA-induced PVO, and fluoroquinolone plus rifampicin was most commonly used in a previous study [6].

Shortening the duration of intravenous antibacterial therapy for PVO and the concomitant lengthening of the duration of oral antibacterial therapy may contribute to shorter hospital stays, fewer catheter-related bloodstream infections, and lower healthcare costs, whereas the use of fluoroquinolones as oral antibacterial agents may increase the risk of emergence of multidrug-resistant bacteria [7] and aortic aneurysms or dissection [8]. Thus, alternative drugs should be sought to solve this dilemma.

Cephalexin is a first-generation cephalosporin oral antibacterial agent with extremely high bioavailability (~ 95%) [9]. Although the concentration of cephalexin in bone is 2.5 μg/mL with 1 g cephalexin administration every 6 h [10], the relevance of this concentration to the clinical efficacy for treating bone infection is unknown. Cephalexin is effective against MSSA-induced skin and soft tissue [11] and osteoarticular infections [12] in pediatric patients. Oh et al. showed that oral beta-lactam treatment of adult patients with MSSA-induced PVO did not increase the treatment failure rate compared with intravenous beta-lactam therapy; however, the study used a variety of oral beta-lactams, and the efficacy of cephalexin alone was unknown [5].

In this single-center, retrospective descriptive study, we demonstrated an 87% treatment success on switching to cephalexin after treatment with intravenous antibacterial agents in adult patients with PVO complicated by MSSA bacteremia. Two patients who did not qualify as treatment success had an efficacy score of 3 (inconclusive): with CRP elevation due to concurrent urinary tract infection and a small CRP pre-value in one patient each, and these were not judged as treatment failures for PVO. Thus, cephalexin may be a promising oral antibacterial option after intravenous therapy for PVO with MSSA bacteremia. The results of this study may be applicable to patients with MSSA-induced PVO without bacteremia, as the disease severity is expected to be less than that with bacteremia.

In total, 80% of the patients in this study had paravertebral, epidural, iliopsoas, or other organ abscesses, and this rate was high compared to that of a previous study on MSSA-induced PVO [13], which could be explained by the fact that only patients with PVO with bacteremia were included in the present study. Surgical treatment was performed in 33% of the patients in this study, more than in a previous report [6], which may also be attributed to the high number of cases with concomitant spinal abscesses. Ceftriaxone and cefazolin were used as intravenous antibacterial agents in more than 80% of cases, in compliance with the guideline recommendations [1]. The median duration of intravenous antibacterial therapy was 36 days, and intravenous antibacterial agents were used for at least 21 days. The median total duration of treatment was 86 days, which was longer than the recommended duration in the guideline. This can be explained for three reasons. First, many of the cases in this study were complicated by abscesses. Second, this study includes cases treated before 2015, when Bernard et al. reported that 6 weeks of treatment was beneficial [4]. Third, when long-term oral antimicrobial therapy is given in an outpatient setting, the intervals between visits are often long, depending on the patient’s condition and availability, and the duration of antimicrobial therapy tends to be extended. Cephalexin was discontinued in one patient due to its side effects (elevated liver enzymes, eosinophilia), but the treatment was successful with a response score of 4 at the time of discontinuation. No patient in this study was concomitantly administered rifampicin. An earlier, double-blind, placebo-controlled trial of *S. aureus* infections showed a trend toward increased treatment success with oxacillin or vancomycin plus rifampicin, although the difference was not statistically significant [14]. Retrospective studies of patients with hematogenous PVO also showed a trend toward lower relapse with rifampicin when the causative organism was *S. aureus*; however, no statistically significant difference was demonstrated [15, 16]. These results suggest that it is inconclusive whether concomitant use of rifampicin in the treatment of S. aureus-induced PVO and randomized controlled trials are needed to resolve this. This study did not include patients with infective endocarditis or implant infection; therefore, these results may not necessarily be applicable to the aforementioned patients.

We created a new scale for determining treatment efficacy in PVO. Guideline [1] recommends monitoring inflammatory markers (CRP and ESR) along with clinical response for early detection of treatment failure but does not provide clear criteria for treatment success or failure. Previous studies have used treatment success criteria such as “sustained absence of fever, pain, and inflammatory syndrome (CRP ≤ 10 mg/L) at 1 year after the end of treatment [4]” or “disappearance of all clinical signs and symptoms of vertebral osteomyelitis with no residual disability at the end of treatment [17].” When retrospectively evaluating the efficacy of oral therapy after intravenous treatment, it is necessary to ensure that the treatment is successful at the end of intravenous treatment and to evaluate the success of the treatment at the end of or after completion of oral therapy. Therefore, we did not consider it appropriate to apply previous treatment success criteria to this study. We, therefore, developed a score to comprehensively judge the efficacy of treatment based on three axes: patient symptoms (pain), an inflammatory marker (CRP), and radiographic findings at the end of intravenous treatment as well as at the end of oral treatment, respectively (Table 1). Routine MRI follow-up is not recommended [1]; the treatment success can be determined without imaging studies. In addition, because elevated CRP may not necessarily reflect the disease status of PVO (e.g., other concurrent infections), the presence of other comorbidities that could explain the elevated CRP was incorporated into the score. Although the consistency of this score with previous criteria has not been verified and needs further validation, we believe that this score will help to determine the effectiveness of treatment in patients with PVO.

This study had some limitations. First, the small number of cases and the single-arm nature of the study did not permit a demonstration of the treatment efficacy of cephalexin in comparison with other agents. Second, adverse events are not recorded systematically. However, the prescription of cephalexin should be discontinued when serious side effects occur, and the fact that cephalexin could be administered completely in most cases suggests that cephalexin is relatively safe to use. Third, the follow-up period was short. However, as three-quarters of treatment failures occurred within 4.7 months of diagnosis [18], the risk of relapse after follow-up completion is considered relatively low.

## Conclusion

In patients with bacteremic MSSA-induced vertebral osteomyelitis, completing antibiotic treatment with cephalexin is a reasonable option, even in cases with spinal abscess, if at least 3 weeks of effective intravenous antimicrobial therapy is provided.

## Data Availability

All data generated or analyzed during this study are included in this published article.

## Abbreviations

MSSA: Methicillin-susceptible *Staphylococcus aureus*
PVO: Pyogenic vertebral osteomyelitis
CRP: C-reactive protein
ESR: Erythrocyte sedimentation rate
CT: Computed tomography
MRI: Magnetic resonance imaging
CCI: Charlson Comorbidity Index
IQR: Interquartile range
